# Social media engagement analysis of U.S. Federal health agencies on Facebook

**DOI:** 10.1186/s12911-017-0447-z

**Published:** 2017-04-21

**Authors:** Sanmitra Bhattacharya, Padmini Srinivasan, Philip Polgreen

**Affiliations:** 1 Department of Computer Science, The University of Iowa, Iowa City, IA 52242 USA; 2 Linguamatics Solutions Inc., Westborough, MA 01581 USA; 3 Department of Internal Medicine, The University of Iowa, Iowa City, IA 52242 USA

**Keywords:** Social media mining, Facebook, Engagement analysis, Data mining, Hurdle model, Proportional hazards model, Statistical modeling

## Abstract

**Background:**

It is becoming increasingly common for individuals and organizations to use social media platforms such as Facebook. These are being used for a wide variety of purposes including disseminating, discussing and seeking health related information. U.S. Federal health agencies are leveraging these platforms to ‘engage’ social media users to read, spread, promote and encourage health related discussions. However, different agencies and their communications get varying levels of engagement. In this study we use statistical models to identify factors that associate with engagement.

**Methods:**

We analyze over 45,000 Facebook posts from 72 Facebook accounts belonging to 24 health agencies. Account usage, user activity, sentiment and content of these posts are studied. We use the hurdle regression model to identify factors associated with the level of engagement and Cox proportional hazards model to identify factors associated with duration of engagement.

**Results:**

In our analysis we find that agencies and accounts vary widely in their usage of social media and activity they generate. Statistical analysis shows, for instance, that Facebook posts with more visual cues such as photos or videos or those which express positive sentiment generate more engagement. We further find that posts on certain topics such as occupation or organizations negatively affect the duration of engagement.

**Conclusions:**

We present the first comprehensive analyses of engagement with U.S. Federal health agencies on Facebook. In addition, we briefly compare and contrast findings from this study to our earlier study with similar focus but on Twitter to show the robustness of our methods.

## Background

An increasing percentage of the population uses various social media platforms such as Facebook, Twitter, and Tumblr for reasons varying from casual conversations to debating social issues. Around 68% of U.S. adults use Facebook [1] which has over 180 million daily active users in the U.S. and Canada [2] who spend around 40 min per day on this medium [3]. A recent study by PricewaterhouseCoopers showed that in the United States, 24% of adults post about their health experiences on social media with 16% of them posting reviews of medications, treatments, doctors or health [4]. A survey on social media preference among medical students showed 77% of first year medical students and 80% of graduating medical students use Facebook and prefer online media as their primary source of information [5].

Facebook, the most popular social networking website [1], has invigorated a wide range of health sciences studies. Facebook use for disease surveillance [6] or public health issues [7–9] shows its broad scope for improving public health. Researchers have also used Facebook to address specific health concerns. For example, studies have been conducted to assess Facebook’s potential in engaging smokers in smoking cessation treatment [10] and to evaluate it’s scope in recruitment and retention of young adult American veterans into an online alcohol intervention study [11]. While most Facebook based health studies focus on information dissemination to individual users, surprisingly few have focused on how health agencies are involved in Facebook based communications [12–14]. This paper addresses this gap.

We ask the general question: *How can health agencies be more engaging on social media?* We perceive ‘engagement’ as interactions designed to promote some common goal as seen for example in [15]. In the context of this study the interactions between the U.S. Federal health agencies and Facebook users are meant to promote better healthcare knowledge through successful information dissemination and consumption.

The importance of social media for communicating to a broad audience is well acknowledged in journalism [16], politics [17], marketing [18], entertainment [19], etc. Healthcare organizations such as the Centers for Disease Control and Prevention (CDC) or Food and Drug Administration (FDA) have a crucial responsibility to inform the public of critical pandemic events like the spread of H1N1 [6, 20] or Coronavirus [21] and about drug recalls [22] and sexual health information [23]. Interestingly, while the two organizations differ significantly in the number of Facebook posts they are quite similar in the response (activity/post) generated. Like the CDC, the National Cancer Institute of National Institutes of Health (NIH) also has several thousand posts, but their response is quite low compared to the other two organizations. While it may be that the intent behind a post is to inform rather than to generate a response, differences in engagement are notable. We do not yet understand if there are factors associated with these differences. The nature of public engagement with an organization’s messages is an active focus of research in health sciences and in marketing [24]. This is traditionally studied by surveys of health-information seekers [25, 26]. Studies on engagement can inform organizations about topics of public interest [27] or strategies to increase public reach [28]. In contrast to surveys, our study of engagement on social medial is ‘observational’ where we assess public activities in response to posts by U.S. Federal health agencies.

We address two specific questions with respect to Facebook posts from U.S. Federal health agencies and the responses they generate. First, *which Facebook account and post features are associated with the level of engagement, i.e., level of public response in the form of Facebook activity (likes, shares, comments)?* Second, *which Facebook account and post features are associated with the interval length between an agency’s Facebook post and the last activity it generates?*

We analyze an almost comprehensive set of Facebook posts from 72 Facebook accounts of 24 U.S. Federal health agencies. We explore associations between various features and level of activity using hurdle models. We explore the features related to our second question using survival models. Features we examine include standard ones such as the number of page likes as well as less studied features relating to the semantic content of a post.

## Methods

### Data collection

#### Agencies & accounts

We selected health agencies through the Health and Human Services (HHS) Social Hub website [29] which lists all Facebook accounts affiliated to various U.S. Federal health agencies.

#### Posts & activity

The Facebook Graph API [30] was used to collect all posts from an account’s timeline as of late January 2013. For each post, we recorded its unique identifier, number of likes, shares, comments and other metadata as described below.

### Account and post features

We included features that are generally used in Facebook-based studies [12, 31, 32] as well as those that are seldom considered (see Table 1).

**Table 1:** Facebook features examined

Features	Description
Page likes	# of Facebook users liking a page (log-transformed)
Post type	Classification of the post into six categories such as link, photo, video, etc.
Sentiment	Two scores: one for positivity and the other for negativity
Content (Semantic Groups)	Classification of each post into 15 semantic groups using MTI followed by post-processing. Multiple classes per post allowed.

#### Page likes

The number of page likes shows the number of users endorsing an account. A page like is different from a post like which is considered an engagement activity. Users liking a page receive all posts from the account in their news feeds [33]. It seems reasonable to expect page likes to associate with engagement.

#### Post types

The Facebook Graph API provides information about the type of a particular post. Posts are classified into six self-explanatory categories, namely link, music, photo, question, status (a post is an uncategorized status if it is simply text-based and does not belong to any of the other categories), and video/Adobe’s ShockWave Flash format (SWF).

#### Sentiment

We hypothesize that the sentiment of a Facebook post may be associated with engagement. Perhaps more positive sentiment is linked with greater activity, or maybe the reverse holds. We analyze sentiment using a state-of-the-art lexicon-based sentiment classifier, SentiStrength [34]. SentiStrength has been widely applied to social media postings [35] and has been shown to outperform other lexical classifiers [36]. SentiStrength classifies each Facebook post into positive and negative on a scale of +/−1 (neutral) to +/−5 (extreme).

#### Content

One aspect of Facebook analysis that is often overlooked is post content. We hypothesize that some topics are more attractive to a wider group than others. For example, a post about information dissemination of the outbreak of West Nile virus *(“West Nile virus is a potentially serious illness. What you need to know:* *http://go.usa.gov/r9g4* *”)* generated far more activity compared to a job posting from U.S. Public Health Service Nurses (“National Park Service has a Registered Nurse Manager position open in Yosemite, CA. This position closes on November 19. If interested, please send a cover letter and CV to S**** C**** at email@nps.gov.”).

We use the National Library of Medicine’s Medical Text Indexer (MTI) [37] to assign Medical Subject Headings (MeSH) [38, 39] recommendations to each post. MTI is commonly used to recommend MeSH terms to titles and abstracts of biomedical literature and has been shown to be useful in other domains such as clinical text [40]. As an aside we show a novel application of MTI in the social media domain. The semantic types of the MeSH terms are mapped to the fifteen higher level semantic groups by the National Library of Medicine [41]. For example, the high level semantic group “Disorders” comprises of 12 semantic types, namely, Acquired Abnormality, Anatomical Abnormality, Cell or Molecular Dysfunction, Congenital Abnormality, Disease or Syndrome, Experimental Model of Disease, Finding, Injury or Poisoning, Mental or Behavioral Dysfunction, Neoplastic Process, Pathologic Function, and Sign or Symptom.

### Choice of model

As shown later, around 20% of Facebook posts have zero activity (i.e. they receive no likes, shares or comments). This type of distribution of data where the variance (of activity count) is much greater than the mean implies overdispersed data [42] with zero-inflation [43]. Typically linear models such as Poisson or negative binomial regression are used to model count data. However the zero-inflation and overdispersion (*p* < 0.001) requires using two-part count data models such as the hurdle regression model [44]. Hurdle models have two separate components: a zero-portion used to fit the sizeable portion of zero counts in the data and a count-portion to fit the non-zero counts of the data. The zero-portion models whether a count is zero (no activity) or not using a binomial probability model. The count portion determines the conditional distribution of the non-zero counts using a zero-truncated negative binomial or Poisson model. Previous studies on social media engagement [10, 45–47] have shown the power of hurdle models for modeling data with similar characteristics.

We compared different count data regression models (namely, the Poisson, negative binomial, hurdle Poisson and hurdle negative binomial (HNB)) using standard goodness-of-fit measures. The HNB model had the lowest AIC value (297667.3) compared to the Poisson (1,443,334), negative binomial (304590.7) and hurdle Poisson (1,292,709) models, signifying a better fit. The Vuong statistics signifies that hurdle negative binomial model has a better fit compared to the other models. Our comparison of full and nested models such as hurdle negative binomial and negative binomial using the likelihood ratio test also indicates that the former model fits our data best. Variance inflation factor (VIF) yielded VIF scores for all independent variables in our regression analysis that were within the range of zero to five indicating no multicollinearity issues.

The temporal characteristics of a post are also of interest. We use methods from survival analysis [48], the branch of statistics dedicated to modeling such temporal behavior. Similar to other social media based studies [49, 50], we use the Cox proportional hazards regression model [51], specifically, to predict how the different features (see Table 1) associate with the time duration between the Facebook post and the last activity in response.

## Results

### Agencies & accounts

Seventy two Facebook accounts corresponding to 24 health agencies were identified. Seventeen are NIH division such as NIH/NIDA, NIH/NIMH and NIH/NICHD. Some agencies have quite a few accounts such as NIH/NLM (6 accounts: Women’s_Health_Resources, NLM_4_Caregivers, etc.), CDC (10 accounts: CDC_Tobacco_Free, Health_Hazard_Evaluation_Program, etc.), OS (16 accounts: HealthCare.gov, Medical_Reserve_Corps, etc.) while several others have just one account such as ACF, FDA, NIH/NCCAM, etc. Table 2 lists the various agencies, the number of accounts for each and of accounts.

**Table 2:** Agencies and accounts on Facebook

Agency	Name	# accounts	Examples of accounts
ACF	Administration for Children & Families	1	Child_Welfare_Information_Gateway
AoA	Administration on Aging	2	Administration_on_Aging, etc.
CDC	Center for Disease Control & Prevention	10	CDC_Tobacco_Free, etc.
FDA	U.S. Food & Drug Administration	1	U.S._Food_and_Drug_Administration
HRSA	Health Resources & Services Administration	2	Health_Resources_and_Service_Administration_(HRSA), etc.
NIH	National Institutes of Health	8	Fogarty_International_Center, etc.
NIH/NCCAM	National Center for Complementary & Alternative Medicine	1	National_Center_for_Complementary_and_Alternative_Medicine
NIH/NCI	National Cancer Institute	3	National_Cancer_Institute, etc.
NIH/NEI	National Eye Institute	1	National_Eye_Health_Education_Program_(NEHEP)
NIH/NHGRI	National Human Genome Research Institute	1	National_DNA_Day
NIH/NHLBI	National Heart, Blood & Lung Institute	4	National_Heart,_Lung,_and_Blood_Institute_(NHLBI), etc.
NIH/NIAID	National Institute of Allergy & Infectious Diseases	1	National_Institute_of_Allergy_and_Infectious_Diseases_(NIAID)
NIH/NIAMS	National Institute of Arthritis & Musculoskeletal & Skin Diseases	2	National_Institute_of_Arthritis_and_Musculoskeletal_and_Skin_Diseases_Labs, etc.
NIH/NICHD	National Institute of Child Health and Human Development	1	Eunice_Kennedy_Shriver_National_Institute_of_Child_Health_and_Human_Development
NIH/NIDA	National Institute of Drug Abuse	2	Drug_Facts, etc.
NIH/NIDDK	National Institute of Diabetes and Digestive and Kidney Diseases	3	National_Diabetes_Education_Program_(NDEP), etc.
NIH/NIEHS	National Institute of Environmental Health Sciences	1	National_Institute_of_Environmental_Health_Sciences
NIH/NIGMS	National Institute of General Medical Sciences	1	National_Institute_of_General_Medical_Sciences
NIH/NIMH	National Institute of Mental Health	1	National_Institute_of_Mental_Health
NIH/NINDS	National Institute of Neurological Disorders and Stroke	1	Know_Stroke
NIH/NLM	National Library of Medicine	6	Women’s_Health_Resources, etc.
NIH/OBSSR	NIH Office of Behavioral and Social Sciences Research	1	The_Office_of_Behavioral_and_Social_Sciences_Research_(OBSSR)
OS	Office of the Secretary	16	Best_Bones_Forever!, etc.
SAMHSA	The Substance Abuse & Mental Health Services	2	Disaster_Distress_Helpline, etc.
Grand Total		72	

As shown in Table 3, a total of 45,862 posts were collected from the timelines of the 72 accounts. Twenty percent (8986 posts) had no likes, shares or comments i.e. no activity, (9889 (21.5%) posts had no likes, 31,699 (69.1%) had no shares and 30,160 (65.7%) had no comments). Only 2245 posts (4.8%) had 100 or more total shares, likes and comments (total activity = 547,476, mean = 243.8; the highest number of likes, shares and comments for a post were 8436, 1070 and 7552, respectively). The remaining three-fourths (34,631) of posts fell between these ranges (total activity = 513,521, mean = 14.8). In raw numbers the Office of the Secretary (OS) had the highest number of posts (9158) with most (7925) being liked, shared or commented. The CDC with the second highest number of posts (7313) gets the most activity on aggregate (407,910) as well as per post (55.78). The NLM had the highest number and highest percentage of posts with no activity (1695, 42%).

**Table 3:** Posts and activities per agency on Facebook

Agency	#posts	#posts with zero activity	# posts with atleast one activity	# likes	# shares	# comments	# total activity	# activity per post	# activity per non-zero activity post
ACF	372	21 (5.65%)	351 (94.35%)	2235	647	265	3147	8.46	8.97
AoA	1878	320 (17.04%)	1558 (82.96%)	5138	3381	363	8882	4.73	5.70
CDC	7313	1149 (15.71%)	6164 (84.29%)	253,607	118,644	35,659	407,910	55.78	66.18
FDA	538	119 (22.12%)	419 (77.88%)	12,008	6321	6085	24,414	45.38	58.27
HRSA	2456	609 (24.8%)	1847 (75.2%)	8203	1306	2092	11,601	4.72	6.28
NIH	2831	738 (26.07%)	2093 (73.93%)	27,391	10,012	1985	39,388	13.91	18.82
NIH/NCCAM	659	79 (11.99%)	580 (88.01%)	5803	2338	510	8651	13.13	14.92
NIH/NCI	3455	585 (16.93%)	2870 (83.07%)	27,685	4429	5475	37,589	10.88	13.10
NIH/NEI	447	87 (19.46%)	360 (80.54%)	1799	1860	86	3745	8.38	10.40
NIH/NHGRI	417	25 (6%)	392 (94%)	5226	1613	409	7248	17.38	18.49
NIH/NHLBI	3510	524 (14.93%)	2986 (85.07%)	82,420	26,606	6078	115,104	32.79	38.55
NIH/NIAID	632	114 (18.04%)	518 (81.96%)	2811	383	181	3375	5.34	6.52
NIH/NIAMS	414	44 (10.63%)	370 (89.37%)	1165	128	63	1356	3.28	3.66
NIH/NICHD	332	40 (12.05%)	292 (87.95%)	762	192	48	1002	3.02	3.43
NIH/NIDA	1657	177 (10.68%)	1480 (89.32%)	13,772	11,423	1232	26,427	15.95	17.86
NIH/NIDDK	1720	451 (26.22%)	1269 (73.78%)	4702	1239	785	6726	3.91	5.30
NIH/NIEHS	148	47 (31.76%)	101 (68.24%)	287	90	41	418	2.82	4.14
NIH/NIGMS	236	53 (22.46%)	183 (77.54%)	1191	222	166	1579	6.69	8.63
NIH/NIMH	427	23 (5.39%)	404 (94.61%)	13,130	6574	1752	21,456	50.25	53.11
NIH/NINDS	83	17 (20.48%)	66 (79.52%)	427	121	86	634	7.64	9.61
NIH/NLM	4076	1695 (41.58%)	2381 (58.42%)	24,280	5861	1903	32,044	7.86	13.46
NIH/OBSSR	188	75 (39.89%)	113 (60.11%)	212	55	26	293	1.56	2.59
OS	9158	1233 (13.46%)	7925 (86.54%)	172,550	57,372	28,281	258,203	28.19	32.58
SAMHSA	2915	761 (26.11%)	2154 (73.89%)	25,657	11,059	3089	39,805	13.66	18.48
Total	45,862	8986 (19.59%)	36,876 (80.41%)	692,461	271,876	96,660	1,060,997	23.13	28.77
Median	645.5	116.5	549	5514.5	2099	647.5	8766.5	8.42	11.75
Mean (SD)	1910.92 (2327.30)	374.42 (459.42)	1536.50 (1947.75)	28852.54 (60566.59)	11328.17 (25970.74)	4027.50 (8879.44)	44208.21 (94905.29)	15.24 (15.67)	18.29 (18.17)

Table 4 shows the top 10 accounts ranked by activity per post. We note, for example, that one of the six NLM Facebook accounts is in the top 10 list. Let’s Move affiliated to the Office of the Secretary has the highest activity per post (246.2) when excluding posts with no activity. CDC’s official account, with the most number of posts (2867), also leads in total number of activities (285,347).

**Table 4:** Top 10 accounts with most activity per Facebook post

Account (Agency)	# posts	# posts with non-zero activity	# posts with zero activity	# likes	# shares	# comments	# total activity	# activities per non-zero activity post
Let’s_Move (OS)	457	446 (97.59%)	11 (2.41%)	73,144	23,535	13,117	109,796	246.18
StopBullying.Gov (OS)	173	168 (97.11%)	5 (2.89%)	21,882	9583	4788	36,253	215.79
Million_Hearts (CDC)	488	432 (88.52%)	56 (11.48%)	36,041	13,515	2204	51,760	119.81
CDC_Tobacco_Free (CDC)	457	317 (69.37%)	140 (30.63%)	15,315	17,355	1803	34,473	108.75
CDC (CDC)	2867	2667 (93.02%)	200 (6.98%)	177,302	78,890	29,155	285,347	106.99
The_Heart_Truth (NIH/NHLBI)	1056	879 (83.24%)	177 (16.76%)	61,843	21,387	3733	86,963	98.93
National_Institutes_of_Health_(NIH)	427	408 (95.55%)	19 (4.45%)	17,522	8885	947	27,354	67.04
U.S._Food_and_Drug_Administration (FDA)	538	419 (77.88%)	119 (22.12%)	12,008	6321	6085	24,414	58.27
National_Institute_of_Mental_Health (NIH/NIMH)	427	404 (94.61%)	23 (5.39%)	13,130	6574	1752	21,456	53.11
NCBI_-_National_Center_for_Biotechnology_Information (NIH/NLM)	298	260 (87.25%)	38 (12.75%)	9658	1930	619	12,207	46.95

### Account and post features

#### Page likes

Table 5 shows the top 10 accounts with the most page likes. The CDC has the highest number of page likes (241,342) followed by Let’s_Move (115,940).

**Table 5:** Facebook page likes

Account	# page likes
CDC	241,342
Let’s_Move	115,940
Million_Hearts	53,728
StopBullying.Gov	49,721
U.S._Food_and_Drug_Administration	43,240
NCBI_-_National_Center_for_Biotechnology_Information	43,201
National_Institutes_of_Health_(NIH)	35,054
The_Heart_Truth	34,012
National_Institute_of_Mental_Health	32,484
CDC_en_Español	20,923

#### Post types

Table 6 shows the various types of post as well as their counts. Links are the most common (28,830) while questions are the least common (74).

**Table 6:** Count of various post types

Post type	# posts
link	28,830 (62.8%)
status	9121 (19.8%)
photo	6428 (14.1%)
video/swf	1333 (2.9%)
music	76 (0.2%)
question	74 (0.2%)

#### Sentiment

In Table 7, we see that Facebook posts are generally positive (percentage of moderate to extreme positive is 61.89% while for negative this percentage is 47.04%).

**Table 7:** Distribution of positive and negative sentiments for Facebook posts on a 5-point scale

Sentiment-level	# of positive posts	# of negative posts
neutral	17,477 (38.11%)	24,281 (52.94%)
moderate-medium	22,846 (49.81%)	10,426 (22.73%)
medium	4625 (10.08%)	5267 (11.48%)
medium-extreme	905 (1.97%)	5673 (12.37%)
extreme	9 (0.02%)	215 (0.47%)
Total	45,862	45,862

#### Content

Table 8 shows the 15 semantic groups and their prevalence in our Facebook dataset. Note that a particular post can be classified into multiple semantic groups. ‘Concepts & Ideas’ is the most prevalent, 54.34% posts contain terms in this group. ‘Devices’ and ‘Genes & Molecular Sequences’ are the rarest.

**Table 8:** Semantic groups and their prevalence in the Facebook dataset

Semantic Groups	# posts
Concepts & Ideas	24,922 (54.34%)
Living Beings	22,733 (49.56%)
Geographic Areas	19,891 (43.37%)
Disorders	19,826 (43.22%)
Organizations	19,299 (42.08%)
Activities & Behaviors	15,072 (32.86%)
Physiology	14,158 (30.87%)
Chemicals & Drugs	9549 (20.82%)
Procedures	9223 (20.11%)
Objects	9034 (19.7%)
Phenomena	6784 (14.79%)
Occupations	4367 (9.52%)
Anatomy	3731 (8.13%)
Genes & Molecular Sequences	406 (0.89%)
Devices	364 (0.79%)

### Modeling activity using hurdle model

Table 9 presents results from the hurdle regression model. Regression coefficients in the zero-portion are exponentiated as odds ratios (OR) while the exponentiated regression coefficients in the count portion are treated as incident rate ratios (IRR) [52]. When we interpret the results of a particular variable we consider all other variables to remain constant.

**Table 9:** Results of hurdle negative binomial model for Facebook data. The estimate/coefficient (SE), exponent of coefficient (OR and IRR), z and *p*-values (*p < 0.05, **p < 0.01, ***p < 0.001) are shown

	Zero Portion				Count Portion			
	Estimate (SE)	OR	*z* value	*p*	Estimate (SE)	IRR	*z* value	*p*
(Intercept)	−2.71 (0.47)	0.067	−5.763	***	−5.631 (0.169)	0.004	−33.356	***
Log-transformed page likes	1.102 (0.025)	3.010	43.931	***	1.797 (0.01)	6.033	174.673	***
link	−0.817 (0.462)	0.442	−1.77		0.554 (0.162)	1.741	3.421	***
music	−0.48 (0.57)	0.619	−0.843		0.06 (0.223)	1.062	0.271	
photo	−0.22 (0.464)	0.802	−0.475		1.833 (0.163)	6.253	11.267	***
question	−5.62 (0.659)	0.004	−8.528	***	−0.456 (0.54)	0.634	−0.844	
status	−2.499 (0.462)	0.082	−5.408	***	0.861 (0.163)	2.365	5.28	***
video	−0.388 (0.473)	0.679	−0.82		1.041 (0.165)	2.833	6.302	***
Positive Sentiment	0.16 (0.023)	1.174	7.051	***	0.118 (0.009)	1.126	12.986	***
Negative Sentiment	−0.121 (0.015)	0.886	−7.857	***	−0.068 (0.006)	0.934	−10.692	***
Activities & Behaviors	0.644 (0.031)	1.903	20.605	***	0.06 (0.013)	1.061	4.741	***
Anatomy	0.088 (0.051)	1.092	1.743		0.048 (0.022)	1.049	2.191	*
Chemicals & Drugs	0.112 (0.035)	1.118	3.237	**	0.07 (0.015)	1.073	4.771	***
Concepts & Ideas	0.366 (0.027)	1.441	13.361	***	−0.013 (0.012)	0.987	−1.041	
Devices	0.321 (0.161)	1.378	1.998	*	−0.021 (0.066)	0.980	−0.312	
Disorders	0.329 (0.032)	1.390	10.369	***	−0.035 (0.014)	0.965	−2.514	*
Genes & Molecular Sequences	0.567 (0.199)	1.763	2.85	**	−0.084 (0.06)	0.920	−1.402	
Geographic Areas	0.091 (0.041)	1.095	2.232	*	−0.187 (0.017)	0.830	−10.776	***
Living Beings	0.242 (0.028)	1.274	8.675	***	0.01 (0.012)	1.010	0.787	
Objects	0.212 (0.036)	1.236	5.9	***	−0.117 (0.015)	0.889	−7.769	***
Occupations	0.055 (0.05)	1.057	1.108		−0.232 (0.02)	0.793	−11.472	***
Organizations	−0.35 (0.041)	0.705	−8.468	***	−0.078 (0.018)	0.925	−4.425	***
Phenomena	0.257 (0.041)	1.293	6.25	***	0.144 (0.017)	1.155	8.44	***
Physiology	0.284 (0.031)	1.328	9.13	***	0.034 (0.013)	1.035	2.614	**
Procedures	0.2 (0.036)	1.222	5.597	***	−0.034 (0.015)	0.966	−2.277	*
Log(theta)					−0.172 (0.011)	0.842	−15.005	***

#### Analysis for activity presence

The coefficients of the logit regression in the zero portion of the model indicate how the features relate to crossing the ‘hurdle’ of obtaining at least one activity (i.e. either a like, share or comment).

A unit increase in the log-transformed page likes increase the odds of getting at least one activity by 201% (OR = 3.010), all other variables remaining constant. A unit increase in positive sentiment increases the odds of getting an activity by 17.4% while a unit increase in negative sentiment decrease the odds of getting an activity by 11.4%. Of the various post types, questions or uncategorized status posts are both linked to a decrease in the odds of a post getting an activity by 99.6% and 91.8%, all other variables remaining constant. Other post types are not significantly associated with activity. Twelve of the 15 semantic groups increase the odds of getting an activity with the group ‘Activities & Behavior’ showing the highest increase (90.3%). ‘Organizations’ is the only semantic group that decreases the odds of getting an activity by 29.5%.

#### Analysis for activity abundance

We now analyze the coefficients of the negative binomial regression in the count portion of the hurdle model (Table 9). This allows us to focus on posts that cross the ‘hurdle’ of getting at least one activity.

Given a unit increase in the log-transformed count of page likes, the rate of activity is expected to increase by a factor of 6.033, while holding all other variable in the model constant. For sentiment, a unit increase in positive sentiment increases the rate of activity by a factor of 1.126 while a unit increase in negative sentiment decreases the rate of activity by a factor of 0.934, with all other variables remaining constant. Amongst post types, photos, links, uncategorized status or videos increase the expected rate of activity with photos giving the highest increase by a factor of 6.302 with all other variables remaining constant. Of the 15 semantic groups only five have significant positive association with activity abundance. The semantic group ‘Phenomena’ increases the rate of activity by a factor of 1.155 (highest) followed by ‘Chemicals & Drugs’ which increases the rate of activity by a factor of 1.073. Of the six semantic groups having significant negative associations with the abundance of activity, ‘Occupations’ has the largest decrease with a factor of 0.793. Examples of other groups negatively associated are ‘Objects’, ‘Geographic Areas’ and ‘Organizations’.

#### Analysis across hurdle components

Looking across both components of the hurdle model several features show consistent benefit for engagement. These include numbers of page likes as well as positive sentiment of a post. Emphasizing semantic groups such as Activities & Behavior, Chemicals & Drugs, Phenomena and Physiology correlate with increased engagement. Negative sentiment in posts almost always correlates with lower engagement. So does the semantic group Organizations. Post types such as status or video are not important for crossing the initial hurdle of getting at least one activity but then their presence correlate with higher activity rate.

### Modeling activity life span

The median number of days between a date of posting and date of last activity is zero. Almost 80% of posts have their last activity on the same day as the post date, but there are posts garnering attention for months or even years.

Regression coefficients from the Cox proportional hazards model are exponentiated as hazard ratios (HR) and used in the interpretation of the survival models. It is important to note here that a longer interval is desirable for the time to last activity. Thus features with negative coefficients are beneficial. Interpreting the coefficients is as follows. For continuous variables such as log-transformed counts of page likes, a unit increase in these values may change the time to last activity with all other variables remaining constant. For binary variables (each post type or each semantic group) the time to last activity may increase or decrease based on the presence of a feature compared to its absence in a post.

In Table 10 we find that a unit increase in the number of log-transformed page likes increases the time to last activity by 34.6% with all other variables remaining constant. A unit increase in positive sentiment increases the time to last activity by 2.1% while a unit increase in negative sentiment has no significant association with the time to last activity. Of the various post types, the presence of photos or videos are both linked to an increase in the time to last activity. The other post types are not significantly associated with the time to last activity. Amongst the 15 semantic groups, only eight are significantly related to the time to last activity. Posts containing semantic groups ‘Activities & Behavior’, ‘Concepts & Ideas’, ‘Genes & Molecular Sequences’, ‘Phenomena’ and ‘Procedures’ are positively related by 2.9, 2.3, 13.6, 6.5 and 2.7% respectively. ‘Devices’, ‘Organizations’ and ‘Occupations’ are the only ones that decrease the time to last activity by 14.7, 4.3 and 5.6% respectively.

**Table 10:** Results of Cox proportional hazards model for interval between a Facebook post and its last activity. The Coefficient (SE), hazard ratio (HR), *z* and *p*-values (*p < 0.05, **p < 0.01, ***p < 0.001) for various independent variables are shown

	Interval between Facebook Post & Last Activity			
	Coefficient (SE)	HR	*z*	*p*
Log-transformed page likes	−0.424 (0.008)	0.654	−54.583	***
Link	−0.142 (0.128)	0.868	−1.103	
Music	−0.211 (0.172)	0.810	−1.228	
Photo	−0.435 (0.129)	0.647	−3.377	***
Question	0.221 (0.173)	1.248	1.28	
Status	−0.105 (0.129)	0.900	−0.816	
Video	−0.291 (0.131)	0.748	−2.214	*
Positive Sentiment	−0.022 (0.007)	0.979	−2.989	**
Negative Sentiment	0.007 (0.005)	1.007	1.437	
Activities & Behaviors	−0.03 (0.01)	0.971	−2.925	**
Anatomy	−0.004 (0.017)	0.996	−0.207	
Chemicals & Drugs	−0.011 (0.012)	0.989	−0.935	
Concepts & Ideas	−0.023 (0.01)	0.977	−2.376	*
Devices	0.137 (0.053)	1.147	2.593	**
Disorders	0.012 (0.011)	1.012	1.101	
Genes & Molecular Sequences	−0.146 (0.051)	0.864	−2.876	**
Geographic Areas	−0.004 (0.014)	0.996	−0.295	
Living Beings	0 (0.01)	1.000	0.02	
Objects	0.02 (0.012)	1.020	1.66	.
Occupations	0.042 (0.016)	1.043	2.578	**
Organizations	0.054 (0.014)	1.056	3.846	***
Phenomena	−0.068 (0.014)	0.935	−4.988	***
Physiology	−0.005 (0.011)	0.995	−0.434	
Procedures	−0.028 (0.012)	0.973	−2.296	*

## Discussion

Our results show that there is considerable difference between levels of Facebook use and public engagement among organizations. OS and CDC have the most Facebook posts while NIH/NINDS and NIH/NIGMS have less than 200 posts. In terms of engagement, CDC with more than 7000 posts generates the most Facebook activity among agencies. Overall, less than 5% of Facebook posts get more than 100 total shares, likes or comments. We also found that an account’s page likes have strong positive relationships with Facebook activity. This is in line with previous research where page likes have been used as proxy for engagement with specific health condition pages on Facebook [53]. While it is not an easy task for agencies to increase the number of users liking a page [54], it is still an easy metric to follow. Results also show that the photos, videos or interactive links may increase the likelihood of getting more activities over longer period of time. This is in agreement with previous research findings [31, 55, 56], which show that media content and links are key to engaging Facebook users. Quite surprisingly, question-related posts, which are typically posted to encourage public participation or interaction, are apparently not useful in engaging the public. As observed in previous research [31], it can be argued that while questions might encourage user comments, they are unlikely to encourage likes or shares. Probably the organizations can look into more innovative ways to frame questions that would encourage user engagement. The presence of positive sentiment in posts from these government agencies is associated with higher activity. We speculate that positive posts generate greater readership and thus higher engagement compared to negative posts on Facebook, especially in the healthcare domain. This is in contrast to previous research, albeit in a different domain, which show that users participate more in discussions regarding problems or concerns in political posts with negative affect [57]. Semantic groups have not been previously studied in the context of Facebook activities. We found that posts about activities and behaviors, and phenomenon are positively associated with level of engagement. In contrast, posts about organizations and occupations tend to lower engagement. It may be that such posts are meant to be more informative than engaging.

### Comparison with other studies

With goals similar to this research (i.e. to identify factors associated with engagement), we previously published an article where we analyzed tweets from 130 U.S. Federal health agency Twitter accounts [47]. Nineteen out of the 24 Facebook agencies studied here also had accounts on Twitter. Here we compare and contrast the findings from our previous Twitter-based study to our findings from this study. Comparison of accounts from same agencies but across the two platforms shows that Twitter-based accounts post more than Facebook-based accounts. This is likely due of the relative simplicity of Twitter postings. However, Facebook posts on average get more likes, shares or comments than retweet for tweets. In fact, around 27% of Facebook posts get more than 15 total likes, shares and comments, compared to only 10% of tweets that get more than 15 retweets.

Comparison of the results of the statistical models from the two platforms reveals many interesting findings. As in Facebook, the use of URLs in tweets translates to higher engagement. Interestingly, while positive sentiment in Facebook posts correlate to higher engagement, it has negative or no association with the level of engagement in Twitter. The reasons for this are not quite obvious and we would like to investigate this in future research. In terms of semantic categorization, we find that across both social media platforms posts about activities and behaviors, and phenomenon are positively associated with level of engagement. In contrast, posts about organizations and occupations tend to lower engagement across both platforms. Overall, we find our results to be consistent and our methods to be robust for engagement analysis on Facebook and Twitter.

### Limitations

Our research has a few limitations. First, the social media landscape is extremely dynamic. We captured the number of likes, shares and comments as well as the time to last activity of a Facebook post as a snapshot within this dynamic system. Hence the recorded numbers may have changed over time. While our longitudinal data analysis shows that for four out of five posts all activities are generated on the date of the posting itself, we cannot guarantee that a post won’t gather any activity after months or years. This limitation, however, is bound to affect almost any social media based research conducted at a specific point in time and that uses these counts or similar ones as metrics. Second, our study focused only on U.S. Federal health agencies and thus our findings may not be generalizable to other organizations. While we find ample evidence where our findings mirror those of Facebook studies in other domains (as shown in the Discussion section), we would like to investigate the generalizability of our approach in future studies. Third, the intent behind a post is only known to a posting agency. It could be to encourage discussion or to disseminate information. Engagement may not always be the primary motivation of every post or every agency. Hence our results should not be interpreted as general performance metrics for these agencies. Finally, we studied a specific set of features and their correlation to the extent and duration of engagement. While we included many commonly used features as well as some novel ones in this study, there could be other features such as post frequency [58] or posting time [59] that also have correlation to engagement.

## Conclusion

While some previous studies (referenced earlier) have focused on engagement of health departments at a local level, to the best of our knowledge, we present the first comprehensive analyses of engagement with U.S. Federal health agencies on Facebook. Examination of over 45,000 Facebook posts from 72 Facebook accounts belonging to 24 U.S. Federal health agencies reveals a wide range of activity across these accounts. We find that a very small fraction of the 45,000 posts get more than 100 likes, shares or comments, while one-fifth of posts see no activity at all. Content analyses of the posts show, for example, that the majority of posts contain links and are generally positive in sentiment. Statistical analyses show that the number of page likes of an account is associated with higher engagement. We also find that posts containing media or links and expressing positive sentiment correlate with higher or longer engagement. Depending on their goals and objectives, these findings may be used as recommendations by the U.S. Federal health agencies for communications on Facebook.

## Abbreviations

ACF: Administration for Children & Families
AIC: Akaike information criterion
AoA: Administration on Aging
API: Application program interface
CDC: Center for Disease Control & Prevention
FDA: U.S. Food & Drug Administration
HHS: Health and Human Services
HNB: Hurdle negative binomial
HR: Hazard ratio
HRSA: Health Resources & Services Administration
IRR: Incident rate ratios
MeSH: Medical Subject Headings
MTI: Medical Text Indexer
NCBI: National Center for Biotechnology Information
NCCAM: National Center for Complementary & Alternative Medicine
NCI: National Cancer Institute
NEI: National Eye Institute
NHGRI: National Human Genome Research Institute
NHLBI: National Heart, Blood & Lung Institute
NIAID: National Institute of Allergy & Infectious Diseases
NIAMS: National Institute of Arthritis & Musculoskeletal & Skin Diseases
NICHD: National Institute of Child Health and Human Development
NIDA: National Institute of Drug Abuse
NIDDK: National Institute of Diabetes and Digestive and Kidney Diseases
NIEHS: National Institute of Environmental Health Sciences
NIGMS: National Institute of General Medical Sciences
NIH: National Institutes of Health
NIMH: National Institute of Mental Health
NINDS: National Institute of Neurological Disorders and Stroke
NLM: National Library of Medicine
OBSSR: NIH Office of Behavioral and Social Sciences Research
OR: Odds ratio
OS: Office of the Secretary
SAMHSA: The Substance Abuse & Mental Health Services
SD: Standard Deviation
SE: Standard Error
SWF: ShockWave Flash format
URL: Uniform Resource Locator
VIF: Variance inflation factor
